# Potential health risks of complementary alternative medicines in cancer patients

**DOI:** 10.1038/sj.bjc.6601560

**Published:** 2004-01-20

**Authors:** U Werneke, J Earl, C Seydel, O Horn, P Crichton, D Fannon

**Affiliations:** 1 Homerton Hospital, East Wing, Department of Psychiatry, Homerton Row, London E9 6SR, UK; 2 Centre for the Economics in Mental Health, Institute of Psychiatry, De Crespigny Park, London SE5 8AJ, UK; 3 Pharmacy Department, Royal Marsden Hospital, Downs Road, Sutton SM2 5PT, UK; 4 Pharmacy Department, Royal Marsden Hospital, Downs Road, Sutton SM2 5PT, UK; 5 Academic Department of Psychological Medicine, King's College School of Medicine and Dentistry and Institute of Psychiatry, De Crespigny Park, London SE5 8AF, UK; 6 Department of Psychological Medicine, Royal Marsden Hospital, Downs Road, Sutton SM2 5PT, UK; 7 Maudsley Hospital & Division of Psychological Medicine, Institute of Psychiatry, Denmark Hill London SE5 8AZ, UK

**Keywords:** complementary alternative medicines, herbal remedies, supplements, cancer, risks, echinacea

## Abstract

Many cancer patients use complementary alternative medicines (CAMs) but may not be aware of the potential risks. There are no studies quantifying such risks, but there is some evidence of patient risk from case reports in the literature. A cross-sectional survey of patients attending the outpatient department at a specialist cancer centre was carried out to establish a pattern of herbal remedy or supplement use and to identify potential adverse side effects or drug interactions with conventional medicines. If potential risks were identified, a health warning was issued by a pharmacist. A total of 318 patients participated in the study. Of these, 164 (51.6%) took CAMs, and 133 different combinations were recorded. Of these, 10.4% only took herbal remedies, 42.1% only supplements and 47.6% a combination of both. In all, 18 (11.0%) reported supplements in higher than recommended doses. Health warnings were issued to 20 (12.2%) patients. Most warnings concerned echinacea in patients with lymphoma. Further warnings were issued for cod liver/fish oil, evening primrose oil, gingko, garlic, ginseng, kava kava and beta-carotene. In conclusion, medical practitioners need to be able to identify the potential risks of CAMs. Equally, patients should be encouraged to disclose their use. Also, more research is needed to quantify the actual health risks.

The use of complementary alternative medicines (CAM) is well documented (Ernst and Cassileth, 1999). These are either used on their own (alternative) or in addition to conventional medicine (complementary) (Zimmerman and Thompson, 2002). This is particularly common in patients suffering from chronic disorders such as cancers and their associated physical and psychological problems. Depending on the definition and inclusion criteria chosen, estimates range from 7 to 64% in the reported prevalence of CAM use in cancer patients (Ernst and Cassileth, 1998). More recent studies have reported an even higher prevalence of between 70 and 80% (Richardson *et al*, 2000; Bernstein and Grasso, 2001; Ashikaga *et al*, 2002). The nature of CAMs used, for example, vitamins and other supplements, herbal remedies, physical and psychological treatments, also varies greatly (Risberg *et al*, 1998; Richardson *et al*, 2000; Sparber *et al*, 2000; Bernstein and Grasso, 2001; Ashikaga *et al*, 2002).

Patients with chronic illnesses who seek alternative therapies are likely to use conventional medicine regularly and simultaneously. However, they may not always inform their doctor of the concomitant use of alternative medicine. For instance, a study of Eisenberg and co-workers in the US showed that 96% of alternative-medicine users also sought a conventional medicine provider for at least one medical condition. In all, 28% used alternative medicine for the same medical condition, and 72% did not inform their physician (Eisenberg *et al*, 1993; Kessler *et al*, 2001). The reasons for CAM use have been widely investigated. Patients often wish to combine conventional and CAM approaches to improve their quality of life, to counter side effects, to achieve a sense of control and to match their life style with their world view (Austin, 1998; Sparber *et al*, 2000; Kessler *et al*, 2001).

However, the use of CAM and especially of herbal remedies and supplements is not without problems. Unconventional cancer therapies such as Laetrile, Essiac and coenzyme Q10 may not be effective (Ernst and Cassileth, 1999). Furthermore, CAMs have potentially dangerous side effects and interactions with conventional treatments. For instance, garlic and cod liver oil have anticoagulant effects (Fugh-Berman, 2000), and remedies acting on the cytochrome P450 system such as St John's wort, may interact with hormones, antibiotics and chemotherapeutic agents (Izzo and Ernst, 2001).

Many reviews of the potential dangers have been published, but clinical accounts are mostly confined to individual case reports of adverse events (Ernst, 1998). The purpose of this survey was to prevent potential health risks, which CAM users might encounter. We aimed to establish the type, frequency and pattern of herbal medicine and supplement use in a sample of cancer patients and to identify and quantify the potential for adverse side effects or drug interactions with conventional medicines.

## METHODS

We conducted a cross-sectional survey of patients attending the outpatient departments at the Royal Marsden Hospital, a specialist cancer centre using a multiple-choice questionnaire to estimate the presence, frequency and purpose of herbal medicines and supplement use. In addition, respondents were asked whether they had discussed their CAM therapy with their medical practitioners. The questionnaire was piloted on 5% of the sample, and amended as necessary. The completed questionnaires were returned to the Medicines Information Service at the Royal Marsden Hospital pharmacy. There they were scrutinised for potentially serious adverse effects or interactions with prescribed medicines using the web-based and library resources. If the potential for an adverse drug reaction or interaction was detected, the pharmacist (CS) issued a health warning to the patient and treating doctor or GP. The data were entered into a database and analysed descriptively using SPSS version 10. Patients gave written informed consent before participation in the study. The project had received ethical approval from the Royal Marsden Hospital Ethics Committee.

## RESULTS

Of the 500 patients invited to participate, 318 (63.6%) agreed to take part in the study, of whom 60.4% were female. As the study was conducted immediately after consent had been obtained, it was difficult to establish the reason for nonparticipation. However, 65.0% of the nonparticipants stated that the study did not apply to them as they were not taking any CAMs.

Of the patients surveyed, 164 (51.6%) took herbal remedies and/or food supplements. In all, 133 different substances and combinations were recorded. Of these, 16 (9.8%) took CAM in the form of homeopathic preparations. Patients took on average 1.8 (±2.34) supplements; 40.9% took more than one substance and three patients took 10 or more preparations, and 17 (10.4%) only took herbal remedies, 69 (42.1%) only supplements and 78 (47.6%) a combination of both. Among the alternative remedies, Echinacea, evening primrose oil, ginkgo, milk thistle and essiac were most popular (Table 1a

**Table 1:** (a) Alternative remedies taken (*n*=166^a^) (b) supplements and supplement combinations taken (*n*=324^a^)

**Remedy**	***n***	**%**
(a)		
Echinacea	35	21.1
Evening primrose oil	33	19.9
Ginkgo	16	9.6
Milk thistle	11	6.6
Essiac	10	6.0
Chinese remedies (except green tea)	7	4.2
Garlic	7	4.2
St John's wort (Hypericum)	6	3.6
Arnica	5	3.0
Valerian	5	3.0
Bach flower remedies	4	2.4
Green tea	3	1.8
Kava Kava	3	1.8
Siberian Ginseng	3	1.8
Passion Flower	2	1.2
Aloe Vera	2	1.2
Indian remedies incl. turmeric and ginger	2	1.2
Laetrile (vitamin B17)	2	1.2
Panax Ginseng	2	1.2
Wild yam	2	1.2
Golden seal	1	0.6
Grape seed extract	1	0.6
Kelp	1	0.6
Mistletoe (Iscador)	1	0.6
Shark cartilage	1	0.6
Slippery elm	1	0.6

(b)		
Vitamin C/E/combination ACE	53	16.4
Cod liver oil	34	10.5
Selenium	20	6.2
Beta-carotene	7	2.2
Coenzyme Q10 (Ubiquinone)	1	0.3
Germanium	1	0.3
Multivitamins	104	32.1
Other combinations	104	32.1

a 40.9% of patients took more than one CAM.

). Individual supplements included vitamin C, E and a combination of vitamin A, C and E (ACE), cod liver oil, selenium, beta-carotene, coenzyme Q10 and germanium. However, the majority took either multivitamins or other combinations, which were difficult to quantify in detail (Table 1b).

Half of all patients took CAMs for the nonspecific purpose of improving their health or in order to fight cancer, rather than for a specific indication such as boosting their immune system. Most patients took the remedies according to their purported indication, although many of the indications, particularly anticarcinogenic effects, are unproven. Patients with haematological cancer aimed to boost their immune system with echinacea. Patients with breast cancer used cod liver oil for joint pain and evening primrose oil for breast soreness or hormonal disturbances. Milk thistle was taken to detoxify the liver, presumably to counter some side effects of chemotherapy. One patient with lung cancer tried shark cartilage that is supposed to inhibit angiogenesis. In all, 41 (25.0%) patients took substances with psychoactive properties. However, 53 (32.3%) patients were not sure about the purpose of a remedy taken. For further reference, the suggested indications for all the listed remedies are listed in Appendix A.

The pharmacy issued health warnings for 20 (12.2%) patients taking herbal medicines or supplements (Table 2a

**Table 2:** Warnings issued by (a) pharmacy: lymphoma (b) pharmacy: breast cancer (c) pharmacy: other cancers

**Diagnosis**	**CAM taken**	**Other medication**	**Concern**	**Advice given**
(a)				
Non-Hodgkin lymphoma	Echinacea	Rituximab	Stimulation of B lymphocytes which monoclonal antibodies are targeting (Stimpel *et al*, 1984; Luettig *et al*, 1989)	Stop echinacea
			Stimulation of phagocytosis	
			Increased activity and mobility of leucocytes.	
			Induction of macrophages to produce cytokines (TNF, IL-1, interferon beta-2) (Stimpel *et al*, 1984; Luettig *et al*, 1989)	
B-cell lymphoma	Cod liver oil	Warfarin	Cod liver oil: increase of INR with high or changing doses (Fugh-Berman, 2000)	Monitor INR
	Evening primrose oil	Sodium valproate	Evening primrose oil: decrease of seizure threshold; decrease of effectiveness of antiepileptic medication (Miller, 1989)	Discuss evening primrose oil with doctor as unclear whether Sodium valproate was taken for epilepsy
Non-Hodgkin lymphoma	Echinacea		Echinacea: stimulation of immune system as above	Stop both agents
	Kava Kava		Kava Kava: hepatotoxic (Escher *et al*, 2001; Russmann *et al*, 2001; Brauer *et al*, 2003; Humberston *et al*, 2003)	
Lymphoma not specified	Echinacea	Corticosteroids, monoclonal antibodies	Stimulation of immune system as above	Stop echinacea
B-cell lymphoma	Kava Kava, Echinacea		Echinacea: stimulation of immune system as above	Stop both agents
			Kava Kava: hepatotoxic	
Hodgkin's lymphoma	Echinacea		Stimulation of immune system but no interactions with Hodgkin's disease yet reported	Avoid long-term use

(b)				
Breast	Ginseng royal jelly	Bendrofluazide	Ginseng: increases or decreases blood pressure (Natural Medicines Comprehensive Database (2003))	Monitor blood pressure, be aware of allergic potential of royal jelly, patient had been hospitalised with an asthma attack shortly after use, unclear whether related
			Royal jelly: allergic reactions possible if history of asthma or atopy (Leung *et al*, 1997; Thien *et al*, 1996)	
Breast	Siberian ginseng	Antihypertensive therapy	Siberian ginseng: increases or decreases blood pressure (Natural Medicines Comprehensive Database (2003))	Monitor blood pressure
	Goldenseal Germanium		Goldenseal: increases of blood pressure (Natural Medicines Comprehensive Database (2003))	Stop germanium
			Germanium: case reports of renal failure, anaemia, neurological and muscular problems (Tao and Bolger, 1992)	
Breast	Wild yam		Oestrogenic effect (Aradhana *et al*, 1992)	Stop wild yam
Breast	Evening primrose oil, Fish oil	Naproxen	Both: increase INR (Brox *et al*, 1981; Natural Medicines Comprehensive Database (2003))	Report any sign of bleeding
Breast	Kava Kava,		Kava Kava: hepatotoxic	Stop kava kava
Breast	Cod liver oil	Ibuprofen	Increases INR in high doses (Brox *et al*, 1981; Natural Medicines Comprehensive Database (2003))	Report any sign of bleeding
Breast	Beta-carotene		Increases risk of lung and prostate cancer in smokers (The Alpha-Tocopherol, Beta Carotene Cancer Prevention Study Group 1994; Heinonen *et al*, 1998; Patrick, 2000)	Stop beta-carotene
Breast	Milk thistle, Goldenseal	Paclitaxel	Both potentially decrease Paclitaxel metabolism (Zuber *et al*, 2002; Daly and King, 2003; Natural Medicines Comprehensive Database (2003)	Stop both agents

(c)				
Prostate	Ginkgo cod liver oil	Diclofenac	Codliver oil: antithrombotic effect, increases INR (Brox *et al*, 1981; Natural Medicines Comprehensive Database (2003))	Report any sign of bleeding
			Ginkgo reduces platelet adhesiveness and platelet count, increases INR (Fugh-Berman, 2000)	
Ovarian	Coenzyme Q10 (ubiquinone)	Warfarin	Coenzyme Q10: reduces anticoagulant properties of warfarin, has vitamin K like effects	Unable to assess safety of combination, therefore not recommended
	Milk thistle		Milk thistle: inhibits warfarin metabolism (CYP2C9) (Heck *et al*, 2000; Daly and King, 2003; Natural Medicines Comprehensive Database (2003))	
Oesophageal	Garlic	Aspirin, Omeprazole	May increase INR, increased risk of gastro-intestinal haemorrhage (Fugh-Berman, 2000)	Report any sign of bleeding
Testicular	Ginkgo, Garlic, Codliver oil	Aspirin	All may increase INR (Brox *et al*, 1981; Fugh-Berman, 2000; Natural Medicines Comprehensive Database (2003))	Report any sign of bleeding
Endometrial	Milk thistle	Doxorubicin	Potentially decreases doxorubicin metabolism (Kivisto *et al*, 1995)	Stop milk thistle
Ovarian	Laetrile (apricot)		Safety concern because of cyanide contents (Natural Medicines Comprehensive Database (2003))	Advised of risk and discouraged use

). Most concerned the use of echinacea in patients with lymphoma. Owing to its immune system-stimulating activity, Echinacea could have interfered with corticosteroid and monoclonal antibody treatment (Natural Medicines Comprehensive Database, 2003). Further warnings were issued for cod liver/fish oil, evening primrose oil, ginkgo and garlic, all of which have coumarinic constituents, as an interaction with warfarin, aspirin and nonsteroidal anti-inflammatory drugs could lead to an increase in INR (Fugh-Berman, 2000; Natural Medicines Comprehensive Database, 2003). Patients were informed of a potential interference of Siberian Ginseng with antihypertensive therapy (Natural Medicines Comprehensive Database). Kava kava is potentially hepatotoxic (Escher *et al*, 2001; Russmann *et al*, 2001), which has led to voluntary withdrawal of all preparations from the UK market. We also issued a qualified warning to one patient taking beta-carotene, who was known to be an occasional smoker. Beta-carotene may increase the risk of prostate and lung cancer in smokers through enhanced production of beta-carotene oxidation metabolites if they are not neutralised by other antioxidants such as vitamin C and E (Heinonen *et al*, 1998; Patrick, 2000). In addition, 18 (11.0 %) patients reported taking supplements higher than the recommended doses. These included: vitamin C (5), vitamin E (4), multivitamins (3), zinc (3), calcium (2), cod liver oil (2) and one of each of the following: selenium, magnesium, glucosamine, germanium, folic acid, tomato tablets and beta-carotene.

Only 46.3% using CAMs had discussed these with a health-care professional involved in their conventional treatment, and reported that 82.9% of the conventional practitioners gave a favourable or neutral response. Conversely, only 56 (34.1%) had consulted an alternative practitioner. Of these 78.6% had discussed their conventional medicines.

## DISCUSSION

Our survey confirms that there is a high prevalence of herbal medicine and supplement use in cancer patients. A substantial proportion of patients used remedies that have the potential to cause serious adverse reactions or drug interactions. To our knowledge, this survey is the first attempt to identify these potential risks for an actual sample of cancer patients before adverse events have emerged. However, we do not know how these potential risks translate into actual events, and research is required to establish the frequency and seriousness of such side effects and drug interactions. As this study was based on voluntary participation and CAM users seemed to be more likely to participate, we may have overestimated CAM use. However, even if all nonparticipants did not use any form of alternative remedy, the proportion of CAM users would still be 33%. Nonparticipation did not affect the risk estimates, that is, the main area of interest in this study. It was also difficult to draw a clear line between remedies and supplements as these overlap and many patients took combinations.

Although most patients had discussed their use with a health-care professional, there remained a considerable potential for harmful effects. There may be different reasons for this. Medical practitioners may not have the expert knowledge required to deal with the large number of potential risks or may not have the time to do so in routine outpatient clinics. Also, patients may not accept their doctors' opinion and may argue that conventional cancer treatment can be equally toxic. Thus patients may require more education on the benefits of CAMs and their risk management. For instance, patients need to know that for some vitamins, effectiveness is only established when taken in fruit and vegetables but not as supplements (Moertel *et al*, 1985) or that effectiveness of supplements may be confined to specifically selected populations (Blot *et al*, 1993; Russell, 2000). They also need to know that supplements may be associated with adverse events including bleeding and liver failure (Palmer *et al*, 2003) or fail to work, for example, high dose vitamin C (Creagan *et al*, 1979). Only recently, the UK Food Standards Agency has reduced the safe upper limit for many supplements (Food Standards Agency, 2003). Also, the potential for CAM to interact with drugs given during diagnostic procedures or radiotherapy needs to be recognised. For instance, kelp can interact with contrast agents containing iodine, as used in bone and thyroid scanning (Eliason, 1998). Antioxidants binding free radicals or remedies increasing photosensitivity may interfere with radiotherapy (Ernst, 1998).

Our survey highlights the importance for conventional health-care professionals to discuss CAM use with their patients. Clinicians need to be aware of CAM-induced side effects or interactions and identify hazards, advising patients accordingly and avoiding uncritical encouragement of potentially harmful use. Otherwise, prescribers may expose themselves to criticism and possibly litigation (Cohen and Eisenberg, 2002). Equally patients should be encouraged to disclose information about CAMs to health-care professionals. Such discussions need to be conducted sensitively in order to avoid alienating patients who may feel that they have not been taken seriously or have been criticised for using CAM. Also, given that about one-third of the remedies used had psychotropic effects, the question of whether CAM users have special psychological needs should be explored.

Also, research on CAMs and their interactions with conventional medicines needs to keep pace with the development of new cancer therapies. Although in randomised controlled trials the proportion of CAM users should be equal in each trial arm, the trial outcome could theoretically be influenced if a CAM specifically interacts with the trial agent but not with the control medication/placebo.

Doctors will need to devote time to discussing CAM use in outpatient clinics, although the complexities of side effects and interactions may require clinics that are run jointly with a local medicines information and toxicology services that provide access to and interpretation of herbal formularies, reference texts and web-based resources such as Natural Medicines Comprehensive Database (2003) (naturaldatabase.com) and Longwood Herbal Task Force (www.mcp.edu/herbal). Also, pharmacists have a key role in updating physicians and sharing important information gathered from patients with other health-care professionals (Klepser and Klepser, 1999). Service models need to be designed and tested to meet this challenge.

## Appendix A

Postulated effects of CAMs (Natural Medicines Comprehensive Database, 2003; Physician Desk Reference for Herbal Medicines, 2000) are given in Tables A1

**Table A1:** Suggested indication: anticarcinogenic

**Remedy**	**Approved by German regulatory authority (Commission E)**	**Selected other/unproven**	**Suggested mechanism of action**
Coenzyme Q10 (ubiquinone)	—	Inhibition of cancer growth; prevention of cardiotoxicity associated with anthracyclins	Antioxidant
Beta-carotene, vitamin C and E and ACE	—	Inhibition of cancer growth; stimulation of immune system	Antioxidants; Vitamin c and E and ACE can neutralise carcinogenic metabolites of beta-carotene
Essiac	—	Inhibition of cancer growth; stimulation of immune system	Burdock root: prevention of angiogenesis and inhibition of tumour neovascularisation (also contains: sheep sorrel, rhubarb and slippery elm)
Goldenseal	—	Inhibition of cancer growth	Berberine: (isoqinolone alkaloid): inhibition of tumour promoters, inhibition of cancer cells; neutropenia resulting from radio- and chemotherapy gastritis, gastric ulcers and gallbladder disease, diarrhoea
Green tea	—	Cancer prevention; inhibition of cancer growth; nausea and vomiting; diarrhoea; caries prevention	Polyphenols: antioxidant
Laetrile (Vitamin B17, Apricot kernels)	—	Cancer prevention	Amygdalin: cytostatic through cyanide release; balance of vitamin deficiency
Mistletoe (Iscador)	—	Cancer prevention and treatment; stimulation of immune system	Viscotoxins and viscumin (mistletoe lectin): modification of intracellular protein syntheses, stimulation of cytokine production, inhibition of tumour colonisation, induction of cell necrosis (Ernst and Cassileth, 1999)
Selenium	—	Cancer prevention; inhibition of cancer growth	Antioxidant
Shark cartilage	—	Cancer prevention; inhibition of cancer growth	Sphyrnastatin 1 and 2: prevention of angiogenesis and inhibition of tumour neovasculariastion
Turmeric	Dyspeptic complaints; loss of appetite	Cancer prevention; inhibition of cancer growth	Curcuminoids: antioxidant, alteration of cancer cell metabolism, cytotoxicity against human chronic myeloid leukaemia

, A2

**Table A2:** Suggested indication: immune-stimulation

**Remedy**	**Commission E approved**	**Selected unproven other**
Arnica	Topical use: respiratory, oral and cutaneous infections; blunt injuries; boost immune system	—
Echinacea	Respiratory, oral and urinary tract infections; wounds and burns; boost immune system	—

, A3

**Table A3:** Suggested indication: psychoactive

**Remedy**	**Commission E approved**	**Selected unproven other**
Bach flower remedies	—	Nervousness, tension
Ginkgo	Symptomatic relief of organic brain dysfunction; intermittent claudication; vertigo and tinnitus of vascular origin	Boost immune system
Kava Kava	Nervousness and insomnia	—
Panax Ginseng	Lack of stamina and fatigue	—
Siberian Ginseng	Lack of stamina; risk of infections	—
Passion flower	Nervousness and insomnia	—
St John's wort (Hypericum)	Anxiety; depressive moods; topical use; skin inflammations, blunt injuries, wounds and burns	—
Valerian	Nervousness and insomnia	—

and A4

**Table A4:** Suggested indications: other

**Remedy**	**Commission E approved**	**Selected unproven other**
Evening primrose oil	—	Premenstrual problems and menopausal hot flashes; mastalgia neurodermitis and atopic eczema
Wild yam	—	Dysmenorrhoea and cramps; postmenopausal symptoms, e.g. vaginal dryness; rheumatic conditions; gallbladder colic
Cod liver oil	Arthritis; prevention of coronary heart disease; vision	Cancer prevention; inhibition of cancer growth; hypertension; hypertriglyceridaemia
Kelp		Regulation of thyroid function
Garlic	Arteriosclerosis; hypertension raised level of cholesterol (hyperlipidaemia)	—
Ginger	Loss of appetite; travel sickness; dyspeptic complaints	—
Milk thistle	Dyspeptic complaints	Liver and gallbladder complaints
Slippery elm	—	Gastritis gastric and duodenal ulcers
Grape seed	—	Venous diseases Blood circulation disorders
Aloe vera	—	Wound healing

.
